# Impact of Branched-Chain Amino Acid Supplementation on Postoperative Serum Albumin Recovery in Older Adults with Hip Fracture: A Propensity Score-Matched Study

**DOI:** 10.3390/jcm14238449

**Published:** 2025-11-28

**Authors:** Sang Yoon Kang, Li Loong Loh, Hong Seok Kim, Jeong Joon Yoo

**Affiliations:** 1 Department of Orthopedic Surgery, Seoul National University Hospital, Seoul National University College of Medicine, Seoul 03080, Republic of Korea; 2 Department of Orthopedic Surgery, Traumatology & Rehabilitation, Sultan Ahmad Shah Medical Centre at International Islamic University Malaysia, Kuantan 25200, Pahang, Malaysia

**Keywords:** amino acids, branched-chain, propensity score, nutritional status, hip fractures, serum albumin

## Abstract

**Background/Objectives**: Hip fracture patients commonly exhibit impaired nutritional status, including low serum albumin levels related to sarcopenia, which may affect recovery. This study evaluated the effect of branched-chain amino acid (BCAA) supplementation on serum albumin levels in hip fracture patients. **Methods**: This retrospective analysis was conducted at a single tertiary referral center on a cohort of patients with hip fractures including femoral neck and intertrochanteric fractures who visited the emergency department between January 2022 and November 2023. After propensity score matching, 106 patients were analyzed (BCAA: n = 53; control: n = 53); prior to matching, 67 treated and 110 control patients were eligible. Patients receiving BCAA supplementation were administered three times daily for six weeks postoperatively and they were matched with controls based on clinical and demographic variables. Controls received standard perioperative care without BCAA supplementation, and no placebo was administered. Serum albumin levels were measured before the surgery and 6 weeks postoperatively. After propensity score matching, 53 patients from each cohort were analyzed. The primary outcome was the difference in serum albumin levels; secondary outcomes were the length of hospital stay (LOS), blood transfusions (Tf), and the incidence of delirium. **Results**: The matched cohorts exhibited comparable baseline characteristics. Analysis revealed a significant increase in serum albumin levels among patients who received BCAA supplementation compared to the matched control group. No differences were found in terms of LOS, Tf, and the incidence of delirium during the index admission. **Conclusions**: This preliminary study suggests a potential positive effect of BCAA supplementation on serum albumin levels in patients with hip fractures. Further prospective investigations with larger sample sizes are warranted to validate these findings and elucidate the clinical implications for nutritional support in this population.

## 1. Introduction

Hip fractures in older patients are associated with increased mortality, prolonged functional decline, and substantial socioeconomic burden [1,2]. One-year mortality following hip fracture has been reported as high as 20–30%, and long-term disability is very common [2]. Over the past two decades, advancements in surgical techniques and perioperative management have contributed to some improvements in outcomes; however, the vulnerability of this population still remains [1].

Compromised nutritional status—particularly hypoalbuminemia—is frequently used to predict postoperative complications [3,4,5,6]. Several studies have shown that low serum albumin levels (below 3.0–3.5 g/dL) at admission correlate with higher complication rates and poorer functional recovery [3,4,5,6,7]. Moreover, nutritional assessment tools like the Controlling Nutritional Status score, which incorporates serum albumin, have been validated as independent predictors of postoperative outcomes in this vulnerable group [8]. Malnutrition is highly prevalent in older adults with hip fractures and is consistently associated with worse outcomes, including higher short-term mortality and impaired recovery of mobility and independence. Recent evidence underscores that timely nutritional assessment and support are integral to comprehensive hip fracture care [9].

Sarcopenia frequently coexists with poor nutritional status in this population and is often compounded by vitamin D deficiency, contributing to inferior functional recovery and recurrent events [10,11]. Sarcopenia is increasingly being recognized not just as a comorbidity, but as an unresolved clinical issue following hip fracture, often persisting long after fracture healing [12]. Therefore, targeted nutritional support addressing both low serum albumin levels and sarcopenia should be warranted in these vulnerable patients.

International guidance, such as the 2022 ESPEN practical guideline for geriatrics, recommends routine screening, individualized nutrition care plans, and adequate protein intake for older inpatients [13]. The NICE Hip Fracture guideline embeds nutrition within the multidisciplinary Hip Fracture Programme and emphasizes delirium prevention and continuity of rehabilitation [14].

Major trauma and hip-fracture surgery trigger a hypercatabolic, pro-inflammatory milieu in older patients for weeks to months, contributing to anabolic resistance and delayed recovery of visceral and muscle proteins. In this context, leucine-enriched branched-chain amino acid (BCAA) can stimulate hepatic protein synthesis via the mammalian target of rapamycin (mTOR) C1 signaling pathway. Leucine sensing by Sestrin–mTORC1 has been demonstrated in the liver, and BCAA—especially leucine—promotes hepatic albumin synthesis via mTOR-dependent pathways [15]. Several clinical studies, even in cohorts with hepatic disorders, have reported improvements in serum albumin levels [16,17,18,19,20]. However, the role of BCAA supplementation within the older hip fracture patients remains poorly explored.

This preliminary study aimed to assess the effect of BCAA supplementation in patients with hip fractures. Using propensity score matching (PSM) to balance baseline differences, we examined the magnitude of changes in serum albumin levels as the primary outcome and evaluated length of hospital stay (LOS), blood transfusions (Tf), and the incidence of postoperative delirium as secondary outcomes.

## 2. Materials and Methods

### 2.1. Study Cohort

This retrospective cohort study included 226 consecutive cases of native hip fracture (including femoral neck and intertrochanteric fracture) treated at a single tertiary referral hospital between January 2022 and November 2023. Patients were divided into two groups: the control group consisted of 110 hips treated between January 2022 and March 2023 without BCAA supplementation, and the intervention group consisted of 67 hips treated between April 2023 and November 2023, during which BCAA supplementation was administered three times daily for 6 weeks postoperatively. The study was conducted in accordance with the Declaration of Helsinki and was approved by the hospital’s Institutional Review Board (IRB No. 2403-063-1518).

**Figure 1:**
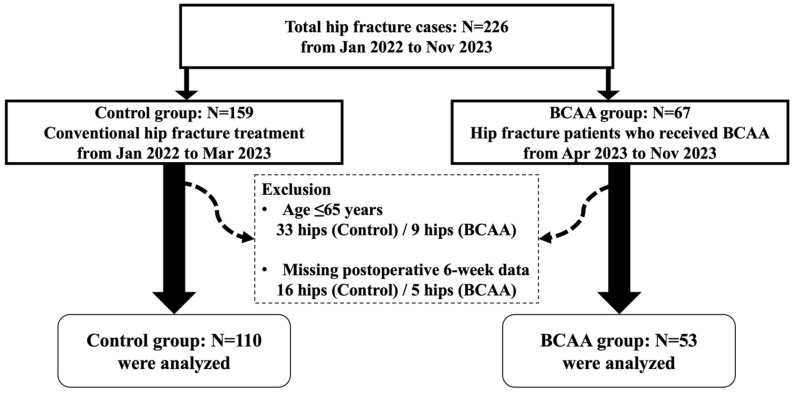
Flowchart of patient selection for the study.

Exclusion criteria included age ≤ 65 years and absence of postoperative 6 weeks laboratory data. After applying these criteria, 110 hips in the control group and 53 hips in the intervention group were included in the final analysis (Figure 1).

All patients were scheduled for standard postoperative follow-ups at approximately 6 weeks and 6 months. And the fracture union was assessed at 6 months. Follow-up completeness through 6 months did not differ between groups.

### 2.2. Intervention (BCAA)

The BCAA product (Livact, Samil Pharmaceutical Co., Ltd., Seoul, Republic of Korea) was administered three times daily for up to 6 weeks postoperatively. Each sachet contained L-leucine 1904 mg, L-isoleucine 952 mg, and L-valine 1144 mg (total BCAA per sachet 4.0 g). Adherence was verified at the 6-week outpatient visit by direct patient (or caregiver) report and empty-sachet check; only participants who completed the full 6-week course were included in the analysis. Adverse events possibly related to BCAA were monitored during follow-up; none were observed.

Controls received standard perioperative care without BCAA supplementation; no placebo was administered.

### 2.3. Baseline Characteristics and Outcome Measurements

All operations were performed by one of two experienced orthopedic surgeons. Baseline demographic variables included age, sex, body mass index (BMI), Charlson Comorbidity Index (CCI), and pre-fracture ambulatory status (Koval grade). Bone mineral density (BMD) was assessed using dual-energy X-ray absorptiometry (DEXA), with the lowest T-score recorded.

Perioperative red blood cell transfusion followed national guidance and hip-fracture literature recommending a restrictive strategy, generally using a hemoglobin threshold around 7.0 g/dL with allowance for clinical judgment in the presence of symptoms or significant comorbidity [21].

Serum albumin was measured preoperatively and at 6 weeks postoperatively; the primary endpoint was the between-group difference in the change in albumin level from baseline to 6 weeks. Secondary endpoints included length of hospital stay, perioperative transfusion, and the occurrence of delirium as documented in the medical record during the index admission.

### 2.4. Statistical Analysis

Propensity score matching was performed to adjust for potential baseline differences between groups. Matching variables included age, sex, BMI, CCI, pre-fracture Koval grade, and lowest DEXA T-score. Propensity scores were generated using logistic regression, and 1:1 nearest-neighbor matching without replacement was applied, resulting in 53 matched pairs (53 patients per group). Continuous variables were compared using the unpaired Student’s *t*-test or Mann–Whitney U test, while categorical variables were analyzed using the chi-square test or Fisher’s exact test, as appropriate. Continuous data are presented as mean ± standard deviation, and categorical data as counts with percentages. The statistical power was calculated at 86% to detect the difference in Δ albumin between the BCAA and control groups after PSM. *p*-value < 0.05 was considered statistically significant. All statistical analyses were performed using IBM Statistical Package for Social Sciences (SPSS) Statistics for Windows version 25.0 (IBM Corp., Armonk, NY, USA).

## 3. Results

### 3.1. Baseline Characteristics

Before PSM, the BCAA group had a significantly higher pre-fracture Koval grade (3.0 ± 2.1 vs. 2.2 ± 1.7, *p* = 0.014) and a lower DEXA T-score (−3.2 ± 1.1, −2.9 ± 1.0, *p* = 0.045) compared with the unmatched control group. Other variables, including age, sex, BMI, and CCI, did not differ significantly between groups. After matching, no significant differences remained between the BCAA group and the matched control group (Table 1).

### 3.2. Serum Albumin Level

In the unmatched analysis, the BCAA group had a higher mean postoperative 6-week serum albumin level compared with the control group (3.7 ± 0.6 g/dL vs. 3.5 ± 0.6 g/dL, *p* = 0.024). After PSM, this difference was not statistically significant (3.7 ± 0.6 g/dL vs. 3.6 ± 0.6 g/dL, *p* = 0.248). However, the change in serum albumin levels from baseline to 6 weeks postoperatively remained significantly greater in the BCAA group compared with the matched control group (0.3 ± 0.5 g/dL vs. 0.03 ± 0.4 g/dL, *p* = 0.008) (Table 2).

Although the mean serum albumin levels at 6 weeks did not differ between groups, the significantly larger Δ-albumin in the BCAA group indicates a steeper within-patient recovery trajectory, with both groups converging to similar absolute levels by 6 weeks. This pattern suggests an earlier biochemical recovery rather than a higher steady-state albumin level at the measured time point.

### 3.3. Clinical Outcomes

The mean LOS was slightly shorter in the BCAA group compared to the matched control group (9.5 ± 6.9 days vs. 10.9 ± 6.9 days, *p* = 0.281). The proportion of patients requiring transfusion in the BCAA group was less than in the matched control group (32.1% vs. 37.7%, *p* = 0.541), with both groups having a median transfusion volume of 800 mL (range, 320–2560 mL in the BCAA group vs. 400–3440 mL in the matched control group, *p* = 0.609). The BCAA group had a lower incidence of postoperative delirium compared to the matched control group (17.0% vs. 18.9%, *p* = 0.800). However, none of these differences in clinical outcomes were statistically significant (Table 3).

## 4. Discussion

Through this study, BCAA supplementation was associated with a significantly greater postoperative increase in serum albumin levels (Δ albumin) than matched controls (0.3 ± 0.5 g/dL vs. 0.03 ± 0.4 g/dL, *p* = 0.008; Table 2). Although absolute albumin at 6 weeks was similar between groups, the larger Δ-albumin in the BCAA group suggests a steeper within-patient recovery trajectory with convergence by 6 weeks under standard care. This result highlights the potential contribution of targeted nutritional support to early anabolic recovery after hip fracture. By balancing key confounders through propensity score matching, our design strengthens the internal validity of this association, while recognizing that the analysis remains observational.

Hip fractures in older adults are associated with substantial morbidity and mortality, often caused by a cascade of complications that stem from pre-existing frailty, malnutrition, and subsequent catabolic stress during recovery. The one-year mortality rate remains high, and many survivors do not regain their pre-fracture function, emphasizing the importance of optimized perioperative management strategies [1,2].

Among prognostic indicators, hypoalbuminemia is a well-established prognostic factor in older hip fracture patients [5]. In our cohort, the mean preoperative serum albumin level was similarly in the lower normal range (3.5 g/dL in the BCAA group and 3.6 g/dL in the matched control group). Given the normal range of serum albumin levels is 3.5–5.5 g/dL, our cohort aligns with prior observations and reflects the high prevalence of suboptimal nutritional status in this population.

After PSM, the Δ albumin was significantly greater in the BCAA group (BCAA 0.3 ± 0.5 g/dL, matched control 0.03 ± 0.4 g/dL, *p* = 0.008), although absolute postoperative albumin levels at 6 weeks did not show significant differences between the groups. This result supports a beneficial effect of BCAA supplementation on the recovery of serum albumin levels during the postoperative period. Comparable findings have been reported in other clinical contexts. For example, BCAA supplementation has helped maintain serum albumin levels in patients with liver cirrhosis or hepatocellular carcinoma [18,19]. To our knowledge, BCAA supplementation has not been previously studied in patients with hip fractures. Our results therefore provide an initial step that its effects could be applicable within the orthopedic trauma area.

Along with hypoalbuminemia, vitamin D deficiency frequently accompanies sarcopenia and impairs both muscle and bone health. The concept of osteosarcopenia has been introduced to represent the confluence of low muscle mass and low bone density [2,22]. Patients with osteosarcopenia represent a particularly frail subgroup, with significantly worse outcomes than those with either condition alone. They are more likely to experience recurrent fractures and delayed recovery, emphasizing the need for targeted nutritional support beyond surgery alone [22].

In this context, nutritional therapy, including BCAA supplementation, has received increasing attention. Their role has been explored in cohorts after gastrointestinal surgery, or in the intensive care unit, with promising results [16,17]. Our findings suggest that BCAAs may provide significant benefits in orthopedic trauma, particularly for older patients with hip fractures, where an increase in Δ albumin could directly affect anabolic recovery. Moreover, one observational study indicates that higher plasma BCAA concentrations are associated with greater hip and spine BMD and lower risk of incident hip fractures [23]. Thus, BCAA supplementation could plausibly support short-term gains in Δ albumin and, over the longer term, bone health.

Although short-term clinical outcomes, including transfusion rates, delirium, and length of hospital stay, were comparable, the significant improvement in serum albumin levels may suggest a potential advantage that could contribute to longer-term outcomes. However, these findings should be interpreted with caution, as long-term outcomes are likely determined by multiple concurrent factors, rather than adjunctive nutritional support alone.

Furthermore, BCAA supplementation was well tolerated. No adverse events were observed in our cohort, and none of the patients discontinued therapy due to intolerance. This aligns with existing literature indicating a favorable safety profile for BCAAs, with few and generally mild side effects reported in other populations [16,17].

Several limitations should be recognized. First, the retrospective design of this study restricts causal conclusions and is prone to information bias. Second, although PSM was used, the lack of randomization means residual selection bias could still exist. Also, confounding related to baseline frailty and bone quality (e.g., Koval grade and DEXA T-score before PSM) cannot be completely excluded. Third, the single-center study design may limit the applicability of the results to other clinical settings. Fourth, we did not assess long-term functional or radiologic outcomes, which would help clarify the clinical importance of recovery of serum albumin levels. Fifth, there is a potential period bias because the intervention group was treated later in the study period, and secular improvements in perioperative care could have affected outcomes despite matching. Finally, this retrospective dataset relied on serum albumin as the sole biochemical nutrition marker; prealbumin and a validated nutrition screening tool (e.g., Mini Nutritional Assessment) were not collected and thus not analyzed. This limits the granularity of baseline nutritional profiling. We plan to incorporate standardized nutrition assessments and additional biomarkers in future prospective studies. Nonetheless, to our knowledge, this is the first study to evaluate the effect of BCAA supplementation in older adults with hip fractures, highlighting the feasibility of targeted nutritional support in this population.

## 5. Conclusions

In conclusion, perioperative branched-chain amino acid (BCAA) supplementation appears to facilitate a more robust postoperative recovery, as indicated by serum albumin levels, in older patients with hip fractures. These preliminary data support the feasibility and biochemical signal of BCAA use in this population. Given the retrospective design and potential period bias, confirmatory multicenter randomized trials are warranted. Future studies should incorporate standardized nutritional assessment and patient-centered outcomes (function, readmissions, mortality) to determine clinical utility and cost-effectiveness.

## Figures and Tables

**Table 1:** Demographic data before and after propensity score matching.

Variables		Control Group		*p*-Value	
	BCAA * Group	Unmatched	Matched	BCAA vs.	BCAA vs.
	(n = 53)	(n = 110)	(n = 53)	Unmatched	Matched
Age (years)	80.5 ± 8.0	80.9 ± 8.3	80.9 ± 8.4	0.776	0.831
Gender				0.998	1.000
Male	13 (24.5%)	27 (24.5%)	13 (24.5%)		
Female	40 (75.5%)	83 (75.5%)	40 (75.5%)		
Body mass index (kg/m^2^)	22.1 ± 3.7	22.3 ± 4.2	21.7 ± 3.3	0.790	0.550
Underweight (<18.5 kg/m^2^)	7 (13.2%)	19 (17.3%)	9 (17.0%)	0.825	0.400
Normal weight (18.5–24.9 kg/m^2^)	37 (69.8%)	68 (61.8%)	37 (69.8%)		
Overweight (25.0–29.9 kg/m^2^)	8 (15.1%)	18 (16.4%)	7 (13.2%)		
Obesity (>30 kg/m^2^)	1 (1.9%)	5 (4.5%)	0 (0%)		
Charlson comorbidity index	5.0 ± 1.8	5.2 ± 2.0	5.2 ± 2.1	0.407	0.490
Pre-traumatic Koval grade	3.0 ± 2.1	2.2 ± 1.7	2.8 ± 2.0	0.014	0.608
Lowest DEXA ** T-score	−3.2 ± 1.1	−2.9 ± 1.0	−3.0 ± 1.1	0.045	0.375
Osteoporosis	38 (71.7%)	79 (71.8%)	40 (75.5%)	0.853	0.693
Osteopenia	14 (26.4%)	27 (24.5%)	12 (22.6%)		

* BCAA: branched-chain amino acid; ** DEXA: dual-energy X-ray absorptiometry.

**Table 2:** Laboratory results before and after propensity score matching.

Variables		Control Group		*p*-Value	
	BCAA * Group	Unmatched	Matched	BCAA vs.	BCAA vs.
	(n = 53)	(n = 110)	(n = 53)	Unmatched	Matched
Preoperative hemoglobin (g/dL)	10.7 ± 2.3	11.5 ± 1.8	11.4 ± 1.9	0.026	0.103
Preoperative albumin (g/dL)	3.5 ± 0.6	3.6 ± 0.4	3.6 ± 0.5	0.353	0.312
Postoperative 6 weeks albumin (g/dL)	3.7 ± 0.6	3.5 ± 0.6	3.6 ± 0.6	0.024	0.248
Δ albumin (g/dL) **	0.3 ± 0.5	−0.03 ± 0.4	0.03 ± 0.4	<0.001	0.008

* BCAA: branched-chain amino acid. ** Δ albumin (g/dL) denotes the change from baseline (preoperative) to postoperatively 6 weeks; values > 0 reflect an increase.

**Table 3:** Comparison of the length of hospital stay, blood transfusion, and delirium incidence of hip fracture patients after surgery.

Variables		Control Group		*p*-Value	
	BCAA * Group	Unmatched	Matched	BCAA vs.	BCAA vs.
	(n = 53)	(n = 110)	(n = 53)	Unmatched	Matched
Length of stay (days)	9.5 ± 6.9	9.4 ± 5.3	10.9 ± 6.9	0.897	0.281
Patients with transfusion (%)	17 (32.1%)	34 (30.9%)	20 (37.7%)	0.880	0.541
Amount of transfusion (mL) (Range)	800 (320–2560)	800 (400–3600)	800 (400–3440)	0.558	0.609
Delirium incidence (%)	9 (17.0%)	17 (15.5%)	10 (18.9%)	0.803	0.800

* BCAA: branched-chain amino acid.

## Data Availability

The data presented in this study are available on request from the corresponding author due to privacy restrictions.
